# The moderate‐intensity continuous exercise maintains renal blood flow and does not impair the renal function

**DOI:** 10.14814/phy2.15420

**Published:** 2022-08-04

**Authors:** Shotaro Kawakami, Tetsuhiko Yasuno, Saki Kawakami, Ai Ito, Kanta Fujimi, Takuro Matsuda, Shihoko Nakashima, Kosuke Masutani, Yoshinari Uehara, Yasuki Higaki, Ryoma Michishita

**Affiliations:** ^1^ Graduate School of Sports and Health Science Fukuoka University Fukuoka Japan; ^2^ Faculty of Sports and Health Science Fukuoka University Fukuoka Japan; ^3^ The Fukuoka University Institute for Physical Activity Fukuoka Japan; ^4^ Division of Nephrology and Rheumatology, Department of Internal Medicine Fukuoka University School of Medicine Fukuoka Japan; ^5^ Department of Rehabilitation Fukuoka University Hospital Fukuoka Japan

**Keywords:** moderate‐intensity continuous exercise, renal function, renal hemodynamics, renal injury

## Abstract

Exercise is restricted for individuals with reduced renal function because exercising reduces blood flow to the kidneys. Safe and effective exercise programs for individuals with reduced renal function have not yet been developed. We previously examined the relationship between exercise intensity and renal blood flow (RBF), revealing that moderate‐intensity exercise did not reduce RBF. Determining the effects of exercise duration on RBF may have valuable clinical applications. The current study examined the effects of a single bout of continuous exercise at lactate threshold (LT) intensity on renal hemodynamics. Eight adult males participated in this study. Participants underwent 30 min of aerobic exercise at LT intensity using a cycle ergometer. Evaluation of renal hemodynamics was performed before and after exercise, in the recovery phase using ultrasound echo. Furthermore, blood and urine samplings were conducted before and after exercise, in the recovery phase. Compared with resting, RBF was not significantly changed immediately after continuous exercise (319 ± 102 vs. 308 ± 79 ml/min; *p* = 0.976) and exhibited no significant changes in the recovery phase. Moreover, urinary kidney injury molecule‐1 (uKIM‐1) level exhibited no significant change immediately after continuous exercise (0.52 ± 0.20 vs. 0.46 ± 0.27 μg/g creatinine; *p* = 0.447). In addition, the results revealed no significant change in urinary uKIM‐1 in 60‐min after exercise. Other renal injury biomarkers exhibited a similar pattern. These findings indicate that a single bout of moderate‐intensity continuous exercise maintains RBF and does not induce renal injury.

## 1 INTRODUCTION

Previous studies have reported that habitual exercise provides numerous benefits to various organs, including the brain, heart, and skeletal muscles (Rowe et al., 2014). In addition, accumulating evidence supports an inverse relationship between physical activity and cardiovascular disease, hypertension, stroke, osteoporosis, type 2 diabetes mellitus, obesity, and colon cancer (Kesaniemi et al., 2001). On the other hand, the number of patients with chronic kidney disease (CKD) has increased over time, and specific management strategies are needed. However, exercise for CKD patients was often restricted because of concerns that exercise may worsen proteinuria and kidney dysfunction (Clorius et al., 1996; Poortmans et al., 1996). In recent years, Yang et al. (2020) reported that exercise training does not aggravate proteinuria in adults with CKD, and exercise could potentially provide a treatment option for CKD patients to enable the suppression of dialysis introduction and extending healthy life expectancy.

Exercise prescription is needed to ensure safe and effective exercise for CKD patients to obtain the beneficial effects from habitual exercise. Exercise prescription for health benefits (e.g., reduction of mobility and/or mortality, improvement of cardiorespiratory fitness) consists of exercise intensity, duration, and frequency. Because renal hemodynamics are strongly influenced by exercise intensity, the decisions about exercise intensity are particularly important for CKD patients. We conducted several previous studies to investigate the relationship between exercise intensity and renal blood flow (RBF) using ultrasound echo (Kawakami et al., 2018; Kotoku et al., 2019). Although para‐aminohippuric acid clearance has been used as a standard method for evaluating RBF (Clorius et al., 1996; Grimby, 1965; Kenney & Ho, 1995; Poortmans et al., 1996), it has the disadvantage of being highly invasive and unable to quickly evaluate RBF. Therefore, we focused on ultrasound echo can be performed noninvasively and is able to quickly evaluate RBF, which place less strain on participants compared with the traditional methods. Then, our results revealed that exercise at moderate intensity, particularly lactate threshold (LT) intensity, caused no decrease in RBF. This findings indicated that LT intensity is a safe exercise intensity (Kawakami et al., 2018; Kotoku et al., 2019). Recently, moderate‐intensity exercise in nondialysis CKD patients has been reported to have beneficial effects (Howden et al., 2012) including antihypertensive effects (Thompson et al., 2019) and improvement of cardiopulmonary fitness (Vanden Wyngaert et al., 2018), physical function (Afsar et al., 2018), and quality of life (Pei et al., 2019). Therefore, the attitudes regarding exercise for CKD patients are shifting from exercise restriction to exercise therapy. Our previous studies revealed that exercise at LT intensity did not decrease RBF, although it remains unclear how continuous exercise at LT intensity affects renal hemodynamics. The appropriate amount of exercise is affected by the exercise intensity and duration, and it is necessary to investigate the influence of exercise duration on renal hemodynamics for clinical practice.

Several studies have shown that the urinary acute kidney injury (AKI) biomarkers such as urinary liver‐type fatty acid‐binding protein (L‐FABP) and kidney injury molecule‐1 (KIM‐1) are increased following exercise (Kosaki et al., 2020; Wołyniec et al., 2018, 2020). These biomarkers are highly sensitive urinary biomarker reflecting the degree of glomerular and/or tubular damage, these results indicate the possibility that glomerular abnormality, tubular hypoxia, and tubulointerstitial damage are caused by exercise. However, the changes of these biomarkers are dependent on the duration and intensity of exercise, most studies investigated changes in AKI biomarkers following high‐intensity exercise involved in reduction in RBF (Kosaki et al., 2020; McDermott et al., 2018; Poussel et al., 2020; Wołyniec et al., 2018, 2020). It is considered that examining the influence of moderate‐intensity continuous exercise on AKI biomarkers is of considerable clinical important.

Therefore, we hypothesized that moderate‐intensity continuous exercise does not impair renal hemodynamics, renal function, and/or induce kidney injury. Thus, we determined variations in renal hemodynamics and the level of burden on the kidneys following continuous exercise at LT intensity by focusing on the relationship between exercise duration and renal hemodynamics.

## 2 MATERIALS AND METHODS

### 2.1 Participants

Eight middle‐aged men were included in this study. The mean ± standard deviation for age, height, and weight were 38 ± 8 years, 176.8 ± 5.7 cm, 68.8 ± 7.3 kg, respectively (Table 1). No participant had any specific underlying diseases (cardiovascular or cerebrovascular disease, receiving dialysis) or a history of associated symptoms and no participants on medications in the current study. All participants underwent a complete medical examination including an electrocardiogram and met all the requirements for participation in this study. Participants avoided strenuous exercise the day before testing and fasted for 8 h prior to testing (drinking water was acceptable) and avoided breakfast, caffeine, exercise on the day. We kept at least a week as a washout period between exercise testing for LT determination and the experimental protocol. Sample size was calculated using G × power version 3.1.9 software (Dusseldolf University, Düsseldorf, Germany) considering the acute effects of moderate‐intensity continuous exercise on renal physiology based on a previous study (Santana et al., 2017) to generate a power of 80% and an alpha risk of 5%. A sample size of eight participants was estimated to be statistically appropriate. All potential risks and procedures were explained to the participants, who provided written informed consent. This study involving human participants was conducted in accordance with the ethical standards of the institutional and national research committee and with the 1964 Helsinki Declaration and its later amendments or comparable ethical standards. This study was approved by the Ethics Committee of Fukuoka University Hospital Approval (No. 21‐02‐M1).

**TABLE 1:** Participant characteristics

Age, years	38 ± 8
Height, cm	176.8 ± 5.7
Weight, kg	68.8 ± 7.3
Body mass index, kg/m^2^	22.1 ± 3.1
Systolic blood pressure, mmHg	113 ± 9
Diastolic blood pressure, mmHg	79 ± 8
Creatinine, mg/dl	0.89 ± 0.04
eGFR, ml/min/1.73 m^2^	79 ± 6
Work load@LT, watts	60 ± 18
VO_2_peak, ml/kg/min	33.9 ± 6.4
VO_2_@LT, ml/kg/min	12.1 ± 3.8
Heart rate@LT, bpm	113 ± 12
%HRR@LT, %	37 ± 14

*Note*: Data are expressed as the mean ± SD.

Abbreviations: eGFR, estimated glomerular filtration rate; HRR, heart rate reserve; LT, lactate threshold; VO_2_peak, peak oxygen uptake.

### 2.2 Exercise testing for determination of optimal exercise intensity

All participants performed incremental maximal exercise test using a cycle ergometer (Lode; Corival, the Netherlands) in advance, and blood was drawn from the ear lobe during exercise to measure blood lactate concentrations. The incremental maximal exercise test consists of a 1‐min warm‐up at 10 W followed by a 20 W increase every minute, as described previously (Kawakami et al., 2018). The discontinuation of incremental maximal exercise test was carried out according to the exercise discontinuation criteria of the American College of Sports Medicine (Liguori et al., 2021). On the basis of our previous findings (Kawakami et al., 2018; Kotoku et al., 2019), we defined exercise at LT intensity that did not show a decrease in RBF as moderate intensity. Regarding exercise intensity for each participant, we determined LT intensity from incremental maximal exercise test. Five technicians assessed the steep increase point of lactate from visual inspection of graphical plots of lactate versus workload during the incremental maximal exercise test. We adopted the mean of three of five measurements of LT intensity, which excluded the maximum and minimum values obtained.

### 2.3 The evaluation of variations in renal hemodynamics during continuous exercise

**FIGURE 1:**
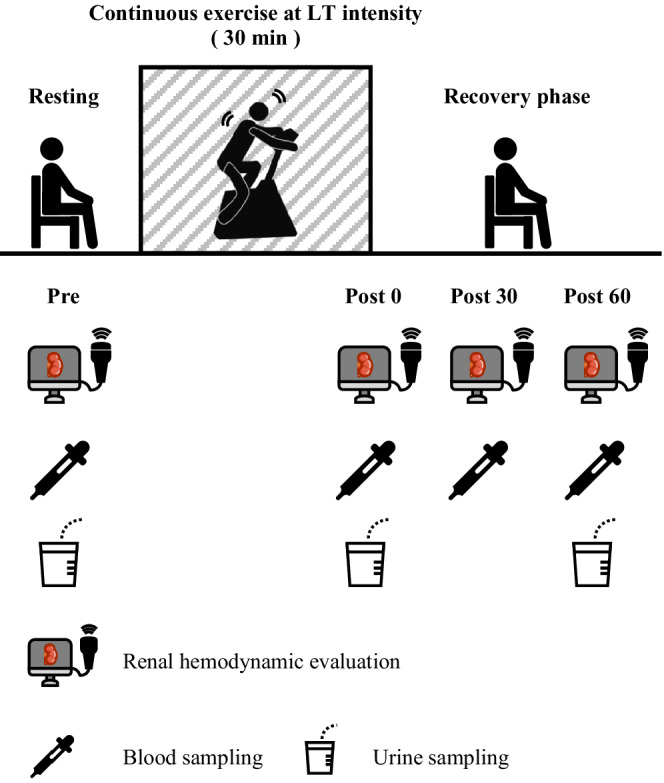
Experimental protocol. Participants underwent 30 min of aerobic exercise at lactate threshold intensity using a cycle ergometer. Renal hemodynamics evaluation was performed in pre and post 0, post 30, and post 60. Blood samples were collected in pre and post 0, post 30, and post 60. Urine samples were taken in pre and post 0, and post 60. Pre, before exercise; post 0, immediately after exercise; post 30, 30‐min after exercise; post 60, 60‐min after exercise.

On arrival at the laboratory, all participants provided urine samples and rested in a sitting position for 5 min (Figure 1). Measurement of renal hemodynamics was then performed using ultrasound echo. Following a 20‐min resting period, blood samples were obtained to determine the blood biochemistry. Participants underwent 30‐min of aerobic exercise at LT intensity using a cycle ergometer. Regarding measurements, renal hemodynamics evaluation and blood sampling were carried out at rest and immediately after exercise, 30‐min after exercise, and 60‐min after exercise. Urine samples were taken at rest and immediately after exercise and 60‐min after exercise. In the current study, variations in renal hemodynamics were evaluated noninvasively using ultrasound echo following long periods of exercise. Furthermore, blood‐ and urine sampling before and after exercise, and in the recovery phase, were obtained to elucidate the mechanism responsible for the regulation of renal hemodynamics and the degree of burden on the kidneys. Participants received hydration freely during and following the experiment.

### 2.4 Evaluation of renal hemodynamics

Evaluation of renal hemodynamics was performed with a pulse Doppler method using a 3.5 MHz convex electronic scanning probe of an ultrasound system (Aplio 300; Toshiba Medical Systems) as described previously (Kawakami et al., 2018). In this study, RBF, peak systolic flow velocity (PSV), end‐diastolic flow velocity (EDV), blood flow velocity (BFV), renal resistive index (rRI), renal pulsatility index (rPI), ratio of PSV to EDV (S/D), and cross‐sectional area (CSA) were determined. First, we placed the probe on the left lateral region of the participant in the seated position and detected the renal artery. We determined the BFV using the pulse Doppler method, and the CSA was determined. RBF was automatically calculated based on BFV and CSA. RBF was calculated as follows: RBF (ml/min) = BFV (cm/sec) × CSA (mm^2^) × 60 (sec). Renal hemodynamic assessment with ultrasound echo has several advantages. For instance, renal hemodynamic assessment with ultrasound echo can noninvasively visualize blood vessels by applying the probe, allowing for repeated measurements over time, leading to diminish the strain on participants. Besides, further benefit of ultrasound echo is that it measures BFV and CSA of renal artery in the kidney independently, enable to determine whether the change in RBF is due to changes in BFV or CSA. Average blood flow rate was defined as the average of three pulsed Doppler waveforms with the participant in the seated position and the probe applied to the left flank. The Doppler incident angle to blood flow direction was measured to within 60° to obtain an accurate measure of BFV. We decided in advance where we would apply the probe and measured at the same position each time. At the time of measurement, the participant held their breath, the probe was placed at the same position, and the measurement was carried out using a high‐resolution echo. The same technician consistently performed the renal hemodynamic assessment throughout this study. The assessment of renal hemodynamics was performed at rest and immediately after exercise, 30‐min after exercise, and 60‐min after exercise.

### 2.5 Measurement of blood and urinary biomarkers

Blood and urine samples were obtained in the morning after 8 h of overnight fasting. A venous blood sample was drawn to measure adrenaline, noradrenaline, plasma renin activity, angiotensin II, aldosterone, creatinine, and cystatin C. In addition, serum creatinine and cystatin C estimation equations (eGFR_cre_ and eGFR_cys_) and filtration fraction (FF) as an indicator of renal function and renal hemodynamics were calculated as follows (Horio et al., 2013; Matsuo et al., 2009):

$${\text{eGFR}}_{\mathrm{cre}}=194\times {\mathrm{Scr}}^{-1.094}\times {\mathrm{age}}^{-0.287},$$

$${\text{eGFR}}_{\mathrm{cys}}={\left(104\times \mathrm{Cys}-C^{-1.019}\times 0.996^{\mathrm{age}}\right)}^{-8},$$

$$\mathrm{FF}\;\left(\%\right)=\text{eGFR}\;\left(\mathrm{ml}/\min/1.73m^{2}\right)/\text{renal plasma flow}\;{\left(\mathrm{RPF}\right)}^{※},$$

$$※\mathrm{RPF}=\mathrm{RBF}\times \text{hematocrit}\;\left(\%\right)$$

=RBF×0.5.

All participants provided a urine sample to examine creatinine, albumin, β2‐microglobulin (β2MG), L‐FABP, and N‐acetyl‐beta‐d‐glucosaminidase (NAG) to determine renal injury in response to exercise. Besides, we measured urinary KIM‐1 in duplicate using a sandwich ELISA kit (Human Urinary KIM‐1 Quantikine ELISA Kit; R&D Systems) to determine the renal injury response to exercise. Moreover, AKI was defined on the Acute Kidney Injury Network (AKIN) criteria for severity of AKI using either parameter: increased serum creatinine or decreased GFR (Mehta et al., 2007). Stage 1 was defined as a 1.5–2‐fold or 0.3 mg/dl increase in serum creatinine concentration from baseline to peak value, whereas stage 2 was defined as a 2–3‐fold increase in serum creatinine concentration. We used the cutoff values with respect to urinary L‐FABP (8.4 μg/g creatinine) and (micro‐) albuminuria (30 mg/g creatinine) to define a kidney injury. Besides, we used the cutoff value for urinary KIM‐1 (31–40 years: 2.14 μg/g creatinine, 41–50 years: 2.24 μg/g creatinine) that has been used to define kidney injury (Pennemans et al., 2013). Each blood sample was centrifuged for 10 min at 1175 *g* at 4°C, and a part of the urine sample was centrifuged for 5 min at 400 *g* at 4°C. Samples were stored at −80°C until analysis. Analyses were performed by a commercial blood and urine company (LSI Medience Corp., SRL Inc.).

### 2.6 Statistical analysis

Experimental data are presented as the mean ± standard deviation. The effects of moderate‐intensity continuous exercise on all outcome measures were analyzed using one‐way analysis of variance with Tukey's post hoc test. Nonparametric data were assessed using the Friedman test. Statistical analyses were performed using Prism version 9.0 (GraphPad Software), and a *p* value of <0.05 was considered to indicate statistical significance.

## 3 RESULTS

### 3.1 Renal hemodynamics following continuous exercise

**FIGURE 2:**
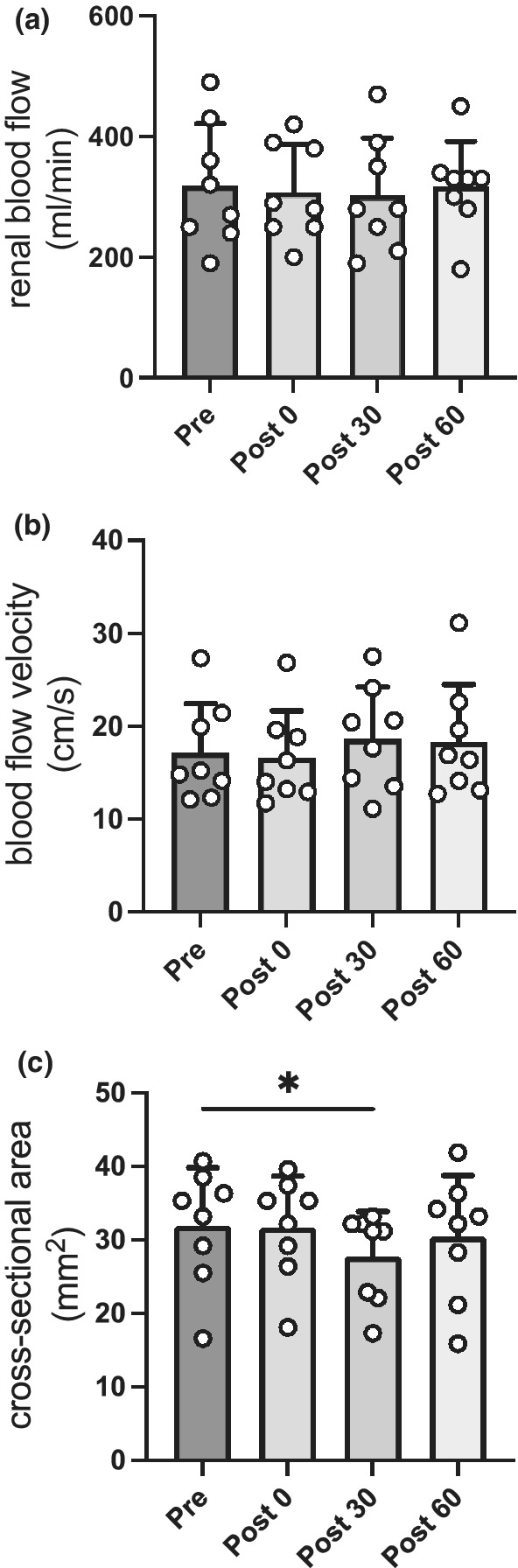
Variations in renal hemodynamics parameters before and after continuous exercise and recovery phase. Changes in (a) renal blood flow, (b) blood flow velocity, and (c) cross‐sectional area in pre and post 0, recovery phases. Data are the mean ± standard deviation. *p < 0.05, compared with values in pre. Pre, *n* = 8; post 0, *n* = 8; post 30, *n* = 8; post 60, *n* = 8. Pre, before exercise; post 0, immediately after exercise; post 30, 30‐min after exercise; post 60, 60‐min after exercise.

**FIGURE 3:**
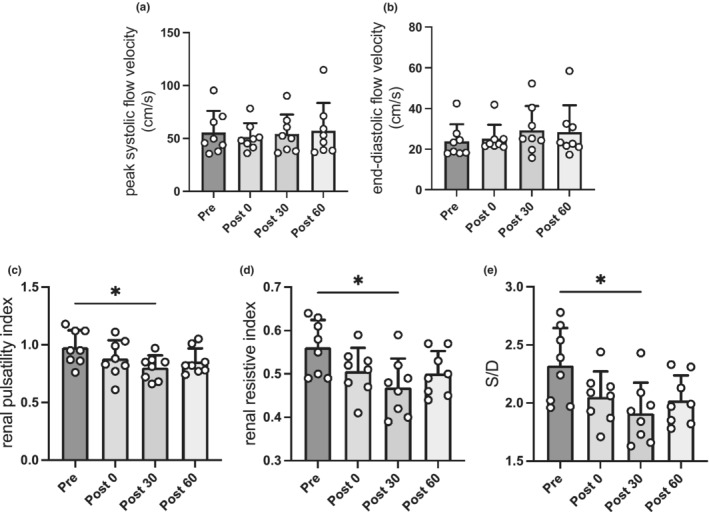
Variations in other renal hemodynamic parameters before and after continuous exercise and recovery phase. Changes in (a) peak systolic flow velocity, (b) end‐diastolic flow velocity, (c) renal pulsatility index, (d) renal resistive index, and (e) S/D in pre and post 0, recovery phase. Data are the mean ± standard deviation. *p < 0.05, compared with values in pre. Pre, *n* = 8; post 0, *n* = 8; post 30, *n* = 8; post 60, *n* = 8. Pre before exercise, post 0, immediately after exercise, post 30, 30‐min after exercise, post 60, 60‐min after exercise. *Renal pulsatility index* (peak systolic flow velocity—end‐diastolic flow velocity)/blood flow velocity, *renal resistive index* (peak systolic flow velocity—end‐diastolic flow velocity)/peak systolic flow velocity, and *S/D* ratio of peak systolic flow velocity to end‐diastolic flow velocity.

RBF immediately after continuous exercise was not significantly changed compared with that before exercise (319 ± 102 vs. 308 ± 79 ml/min, *p* = 0.976). In addition, there was no significant change in RBF in the recovery phase (Figure 2a). RBF is composed of BFV and CSA. In addition, there were no significant changes in BFV at any point compared with before exercise (Figure 2b). However, the CSA at 30 min after continuous exercise was significantly lower than that before exercise (31.9 ± 7.9 vs. 27.8 ± 6.1 mm^2^, *p* = 0.032; Figure 2c). Figure 3 indicates the variations in other renal hemodynamics parameters before and immediately after continuous exercise and in the recovery phase. There were no significant changes in PSV and EDV at any point (Figure 3a,b). Compared with before exercise, the rPI (an indicator of peripheral vascular resistance) showed no significant changes immediately after continuous exercise (0.98 ± 0.15 vs. 0.88 ± 0.16, *p* = 0.329) and showed a significant decrease in 30‐min after continuous exercise (0.98 ± 0.15 vs. 0.80 ± 0.11, *p* = 0.039; Figure 3c). In addition, rRI immediately after continuous exercise exhibited no significant changes (0.56 ± 0.06 vs. 0.51 ± 0.05, *p* = 0.104). However, rRI in 30‐min after exercise significantly decreased compared with before exercise (0.56 ± 0.06 vs. 0.47 ± 0.07, *p* = 0.025; Figure 3d). Moreover, S/D did not significantly change immediately after exercise compared with before exercise (2.32 ± 0.32 vs. 2.05 ± 0.22, *p* = 0.111) and was significantly lower in 30‐min after exercise (2.32 ± 0.32 vs. 1.91 ± 0.26, *p* = 0.024; Figure 3e).

### 3.2 Renal function and injury

**FIGURE 4:**
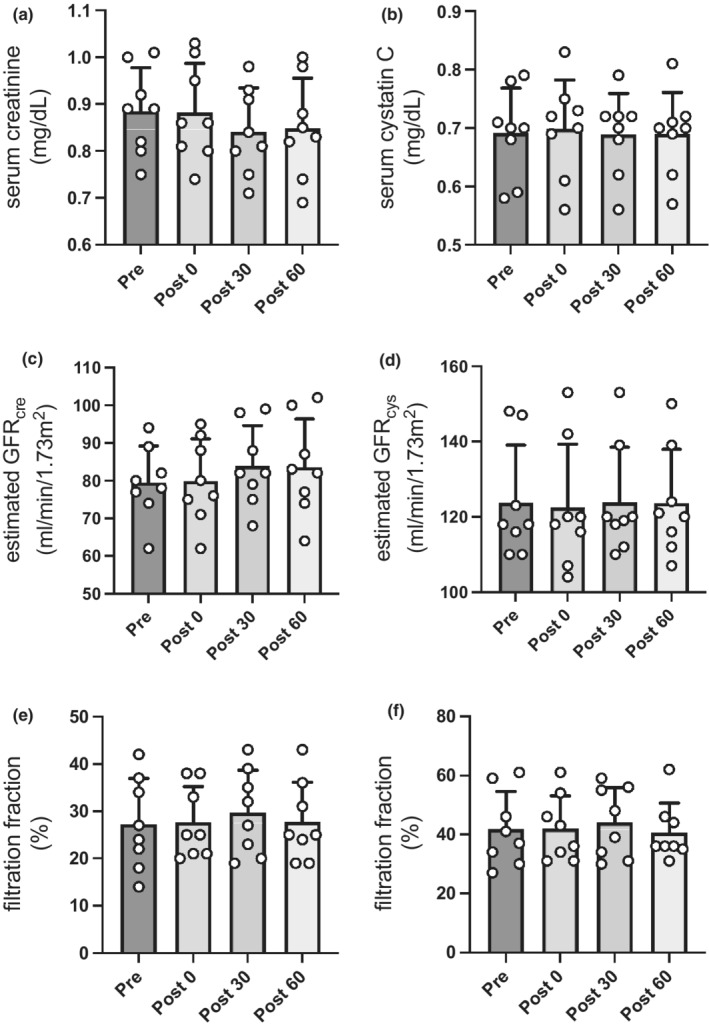
Variations in renal function parameters before and after continuous exercise and recovery phase. Changes in (a) serum creatinine, (b) serum cystatin C, (c) estimated GFRcre, (d) estimated GFRcys, (e) filtration fraction (creatinine), (f) filtration fraction (cystatin C) in pre and post 0, recovery phase. Data are the mean ± standard deviation. *p < 0.05, compared with values in pre. Pre, *n* = 8; post 0, *n* = 8; post 30, *n* = 8; post 60, *n* = 8. Pre, before exercise; post 0, immediately after exercise; post 30, 30‐min after exercise; post 60, 60‐min after exercise. GFR, glomerular filtration rate.

**FIGURE 5:**
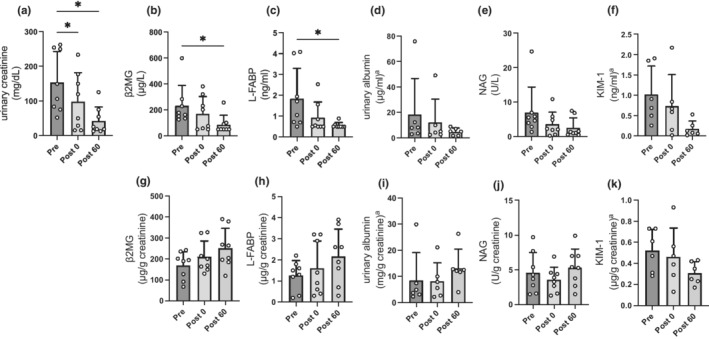
Changes in renal injury parameters before and after continuous exercise and recovery phase. Changes in (a) urinary creatinine, (b) urinary β2MG concentration uncorrected, (c) urinary L‐FABP concentration uncorrected, (d) urinary albumin concentration uncorrected, (e) urinary NAG concentration uncorrected, (f) urinary KIM‐1 concentration uncorrected, (g) urinary β2MG concentration after correction for creatinine, (h) urinary L‐FABP concentration after correction for creatinine, (i) urinary albumin concentration after correction for creatinine, (j) urinary NAG concentration after correction for creatinine, (k) urinary KIM‐1 concentration after correction for creatinine in pre and post 0, post 60. Data are the mean ± standard deviation. *p < 0.05, compared with values in pre. Pre, *n* = 8; post 0, *n* = 8; post 60, *n* = 8. ^a^Data are available as follows: Pre, *n* = 6; post 0, *n* = 6; post 60, *n* = 6. Pre, before exercise; post 0, immediately after exercise; post 60, 60‐min after exercise. β2MG, beta 2 microglobulin; KIM‐1, kidney injury molecule 1; L‐FABP, liver‐type fatty acid‐binding protein; NAG, *N*‐acetyl‐beta‐d‐glicosaminidase.

Regarding variations in renal function with exercise, there were no significant changes in serum creatinine and cystatin C level at any point (Figure 4a,b). Furthermore, eGFR_cre_ and eGFR_cys_ consistently showed no significant changes before and immediately after continuous exercise and in the recovery phase (Figure 4c,d). In addition, continuous exercise at LT intensity had no impact on FFcre and FFcys (Figure 4e,f). Figure 5 shows urinary creatinine, albumin, β2MG, NAG, L‐FABP, and KIM‐1 excretion response to continuous exercise. The urinary creatinine concentrations immediately after continuous exercise and in 60‐min after exercise were significantly decreased compared with those before exercise (*p* = 0.019 and *p* = 0.035, respectively; Figure 5a). Besides, the β2MG levels (233 ± 156 vs. 87 ± 71 μg/L, *p* = 0.026) and L‐FABP levels (1.84 ± 1.45 vs. 0.56 ± 0.14 ng/ml, *p* = 0.012) in 60‐min after exercise were significantly decreased compared with those before exercise (Figure 5b,c). There were no significant changes in albumin levels (18.4 ± 28.4 vs. 12.2 ± 18.3 μg/ml, *p* = 0.745), NAG levels (6.9 ± 7.4 vs. 3.5 ± 3.5 U/L, *p* = 0.137), and KIM‐1 levels (1.02 ± 0.70 vs. 0.74 ± 0.77 ng/ml, *p* = 0.745) before and after exercise (Figure 5d–f). In addition, there were no significant changes in these parameters in 60‐min after exercise. Furthermore, these parameters were adjusted for urinary creatinine concentrations to match the levels of these parameters in accordance with the concentration of urine. After adjusting for urinary creatinine concentrations, there were no significant changes in β2MG levels (169 ± 65 vs. 210 ± 76 μg/g creatinine, *p* = 0.234), L‐FABP levels (1.26 ± 0.70 vs. 1.61 ± 1.28 μg/g creatinine, *p* = 0.668), albumin levels (8.6 ± 10.6 vs. 8.3 ± 7.0 mg/g creatinine, *p* = 0.999), NAG levels (4.6 ± 2.9 vs. 3.6 ± 1.8 U/g creatinine, *p* = 0.325), and KIM‐1 levels (0.52 ± 0.20 vs. 0.46 ± 0.27 μg/g creatinine, *p* = 0.447) before and after exercise (Figure 5g–k). In addition, there were no significant changes in these parameters in 60‐min after exercise.

### 3.3 Biochemical parameters associated with the regulation of renal hemodynamics

**FIGURE 6:**
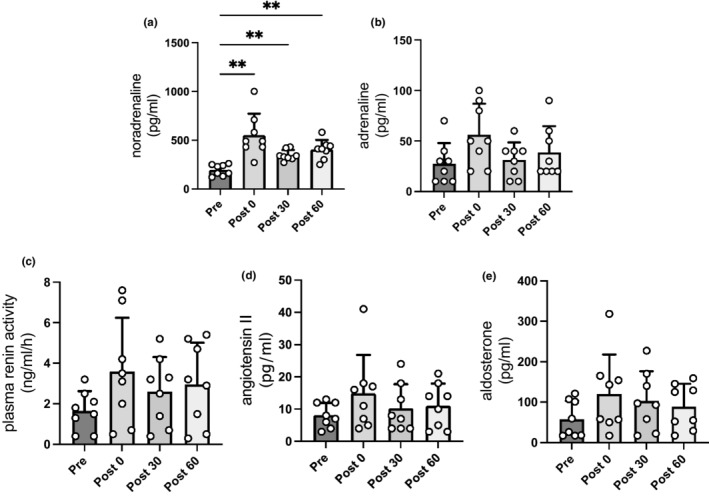
Changes in signaling molecules associated with the regulation of renal hemodynamics before and after continuous exercise and recovery phase. Changes in (a) noradrenaline, (b) adrenaline, (c) plasma renin activity, (d) angiotensin II, and (e) aldosterone in pre and post 0, recovery phase. Data are the mean ± standard deviation. *p < 0.05, compared with values in pre. **p < 0.01, compared with values in pre. Pre, *n* = 8; post 0, *n* = 8; post 30, *n* = 8; post 60, *n* = 8. Pre, before exercise, post 0 immediately after exercise, post 30, 30‐min after exercise, post 60, 60‐min after exercise.

Figure 6 shows the changes in biochemical parameters associated with the regulation of renal hemodynamics before and after continuous exercise and in the recovery phase. Plasma noradrenaline levels were significantly increased after exercise (194 ± 57 vs. 550 ± 221 pg/ml, *p* = 0.009) and remained significantly higher in 60‐min after exercise (194 ± 57 vs. 404 ± 99 pg/ml, *p* = 0.009; Figure 6a). However, there were no significant changes in adrenaline, plasma renin activity, angiotensin II, and aldosterone at any point compared with before exercise (Figure 6b–e).

## 4 DISCUSSION

The current study is the first to reveal variations of renal hemodynamics following continuous exercise at LT intensity using ultrasound echo. The two main findings of this study were as follows: (1) a single bout of continuous exercise at LT intensity maintains RBF, eGFR, and FF and (2) renal injury is not induced following a single bout of continuous exercise at LT intensity.

Many previous studies have indicated a relationship between exercise and RBF (Clorius et al., 1996; Grimby, 1965; Kenney & Ho, 1995; Poortmans et al., 1996). Para‐aminohippuric acid clearance has been used as a standard method for evaluating RBF in most of the previous studies. Although this method provides useful information, it has the disadvantage of being highly invasive and requiring blood and urine sampling and making it difficult to assessment changes in RBF over time. Therefore, several previous studies evaluated RBF using magnetic resonance imaging and ultrasound echo (Chapman et al., 2020; Eckerbom et al., 2020; Khatir et al., 2015; Shimamoto et al., 2017). Assessment of RBF using ultrasound echo can be performed noninvasively and is able to quickly visualize blood vessels, allowing for repeated measurements over time, which place less strain on participants when compared with the traditional methods. Furthermore, the advantage of measurement by ultrasound echo is that it enables the evaluation not only of RBF but also other renal hemodynamic parameters. In the current study, we also used ultrasound echo to determine the detailed renal hemodynamic responses, including RBF, to exercise. We examined the effects of moderate‐intensity continuous exercise on BFV and CSA constituting RBF to explore the effects of exercise on RBF in more detail. BFV exhibited no significant changes before and after exercise and in recovery phase, suggesting that moderate‐intensity continuous exercise had no effect on BFV. In contrast, although the CSA did not significantly change immediately after exercise, a significant decrease was observed in 30‐min after exercise. In addition, we evaluated signaling molecules associated with the regulation of renal hemodynamics to elucidate the mechanism responsible for the regulation of renal hemodynamics. The results revealed that adrenaline, plasma renin activity, angiotensin II, and aldosterone showed no significant changes before and after exercise and in recovery phase, and noradrenaline levels, which exhibited a strong vasoconstrictive effect, were significantly increased after exercise and the recovery phase. Thus, the current findings suggest that increasing noradrenaline following moderate‐intensity continuous exercise contributes to a decrease in CSA. However, no decrease in RBF following moderate‐intensity continuous exercise was observed in this study, suggesting that enhanced sympathetic nervous system activity following moderate‐intensity continuous exercise has some effect on CSA but does not induce a decrease in RBF.

Furthermore, we evaluated FF consisting of eGFR relative to RBF to examine the effect of moderate‐intensity continuous exercise on intrarenal hemodynamics in this study. Therefore, our findings demonstrate that moderate‐intensity continuous exercise caused no decreases in RBF and eGFR, resulting in nonsignificant changes in FF. Several previous studies have examined the impact of exercise on FF and have shown that exercise increases FF (Grimby, 1965; Svarstad et al., 2002). Svarstad et al. (2002) reported that exercise causes a significant increase in FF in CKD patients with hypertension. However, their results also revealed that the administration of enalapril suppresses this increase in FF, suggesting that RAS contributes to the increase in FF with exercise (Svarstad et al., 2002). This study also assessed the effects of moderate‐intensity continuous exercise on plasma renin activity, aldosterone, and angiotensin II, which are indicators of RAS system function, and found that moderate‐intensity continuous exercise caused nonsignificant changes in RAS indicators. Our findings demonstrate that moderate‐intensity continuous exercise does not activate the RAS system, resulting in no significant increase in FF.

Postexercise proteinuria is one of the most common signs observed after exhausting exercise (Ene‐Iordache et al., 2016). Importantly, proteinuria is a marker of renal damage and a predictor of the progress of CKD (Ene‐Iordache et al., 2016). Albuminuria is increased during exercise because of altered permeability of the glomerular membrane and a saturation of the tubular reabsorption process of filtered proteins (Poortmans & Vanderstraeten, 1994). Zambraski et al. (1981) suggest that exercise decreases the glomerular electrostatic barrier and could partially explain the enhanced transglomerular passage of macromolecules. In addition, Poortmans et al. (1988) reported that the reabsorption of albumin and β2MG at the proximal tubules was inhibited by lysine perfusion in humans. The increased filtration fraction is likely to enhance the diffusion process of macromolecules into the tubular lumen, which is one of the causes of albuminuria during exercise. In addition, Poortmans and Vanderstraeten (1994) emphasized that marked albuminuria is observed only after the onset of blood lactate accumulation. Organic acids such as lactic acid and pyruvic acid, which are produced by exercise, may alter glomerular permeability and inhibit the reabsorption of proteins at the proximal tubules. Postexercise proteinuria occurs during exhaustive exercise, suggesting that no significant increases in urinary albumin or β2MG were observed following exercise in this study because we adopted an exercise intensity level (36% VO_2max_) that did not enhance FF and did not increase blood lactate concentration.

Serum creatinine and GFR need to be monitored to define AKI (Mehta et al., 2007). Meanwhile, previous studies have reported that the novel biomarkers of kidney injury are elevated in AKI even before the increase in creatinine and the appearance of albuminuria (Schrezenmeier et al., 2017). Some studies have demonstrated the influence of physical exercise on the levels of AKI biomarkers (Juett et al., 2020; Kosaki et al., 2020; McDermott et al., 2018; Wołyniec et al., 2018, 2020). Previously, Wołyniec et al. (2018) examined changes in urinary neutrophil gelatinase‐associated lipocalin (NGAL) and KIM‐1 after a short period of maximal exercise, reporting that KIM‐1 increased significantly after a very short period of exercise, whereas NGAL did not change. More recently, Kosaki et al. (2020) investigated the acute effects of a short period of maximal exercise on urinary L‐FABP which is a highly sensitive tubular biomarker in adults. The results indicated that short maximal exercise may induce a significant increase in L‐FABP, suggesting that it may lead to acute slightly adverse effects on tubular conditions (Kosaki et al., 2020). Elevated AKI biomarkers following exercise may reflect glomerular and tubular (especially proximal tubules) damage and injury, which could be caused by higher exercise intensity and prolonged exercise duration, producing increased SNS and body temperature, dehydration, and muscle damage. As described above, most previous studies considering the effects of exercise on AKI biomarkers have examined the effects of high‐intensity exercise. In contrast, regarding the effects of moderate‐intensity continuous exercise on renal function and AKI biomarkers, Santana et al. (2017) investigated the acute effects of a 30‐min moderate‐intensity aerobic exercise bout on GFR and albuminuria in nondialysis CKD patients, suggesting that a single bout of moderate‐intensity exercise did not impair renal function. In addition, an investigation by Bongers et al. reported the effects of single versus repetitive bouts of exercise on kidney injury markers in a healthy population. It has been reported that a single bout of prolonged exercise significantly increased NGAL concentration, whereas no changes in KIM‐1 were found (Bongers et al., 2017). However, few studies have examined the effects of a single bout of moderate‐intensity continuous exercise on AKI biomarkers. Safe and effective exercise conditions that do not impair renal function need to be considered for the development of exercise programs to prevent a decrease in renal function. Therefore, in the current study, we examined how moderate‐intensity continuous exercise affects urinary novel AKI biomarkers and revealed no significant changes in urinary NAG, L‐FABP, and KIM‐1 following moderate‐intensity continuous exercise. Our findings suggest that moderate‐intensity continuous exercise does not impair glomeruli or tubules.

Previous studies demonstrated that rRI and rPI, measures of intrarenal hemodynamics derived from ultrasound echo, are associated with albuminuria and AKI (Bellos et al., 2019; Hamano et al., 2008; Yamanaka et al., 2022), and these may be useful tools for examining the detailed renal hemodynamic responses to exercise. The PSV, EDV, and BFV are measured from the blood flow waveform pattern of renal arteries, and the rRI and rPI obtained from the following formula are used as indicators to reflect peripheral vascular resistance rather than the measurement site: rRI = [(PSV−EDV)/PSV], rPI = [(PSV−EDV)/BFV] (Heine et al., 2005; O'Neill, 2014). In the current study, decreases in rRI and rPI were observed in 30‐min after exercise, and S/D also showed a significant decrease (ΔPSV: +1.1% and ΔEDV: +23.3% in 30‐min after exercise; vs. Pre). A recent study reported that renal capillary wedge pressure and peripheral resistance contribute to EDV (Di Nicolò & Granata, 2017). Therefore, the decreases in rRI and rPI following exercise reflect the attenuation of peripheral vascular resistance in the kidneys, suggesting that moderate‐intensity continuous exercise may have beneficial effects on renal circulation. However, a variety of factors have been reported to be involved in these indices, and many of these factors are not fully understood (Di Nicolò & Granata, 2017). Thus, further research is required to explore the influence of exercise on intrarenal hemodynamics.

The current study involved several limitations. First, one of the main limitations of the study was a relatively small sample size, which should be kept in mind when interpreting the results. Second, we examined the acute effects of continuous exercise at LT intensity on renal hemodynamics in this study. We found that a single bout of continuous exercise at LT intensity maintains RBF and does not induce any significant increase in indicators of renal injury. However, the effects of habitual continuous exercise at LT intensity on renal hemodynamics remain unclear. Elucidating the chronic effects of continuous exercise at LT intensity on renal hemodynamics may provide valuable evidence to inform the development of exercise programs for CKD patients. Therefore, we plan to clarify the chronic effects of continuous exercise at LT intensity on renal hemodynamics on the basis of the current results. Third, we thought that the possibility of weight loss (hypovolemia) due to exercise‐induced sweating was low because the room temperature was kept constant and all participants were allowed to hydrate freely throughout the experiment, but we did not evaluate body weight before and after exercise. Finally, it is possible that the renal hemodynamic response to exercise changes depending on the severity of renal functional impairment. However, previous studies have reported an increased risk of cardiovascular disease or mortality with decreased renal function (Go et al., 2004; Nakayama et al., 2011). Thus, we should begin the study with individuals (eGFR ≥60 ml/min/1.73 m^2^) considering safety, so, we examined middle‐aged individuals (38 ± 8 years) with relatively well‐maintained renal function (eGFR ≥60 ml/min/1.73m^2^) in the current study. However, for clinical applications, similar studies should be conducted in nondialysis CKD patients (eGFR <60 ml/min/1.73m^2^) in the future.

To date, appropriate exercise training programs for CKD patients have not been established, and safe and effective exercise conditions that minimize the burden on the kidneys are not fully understood. Elucidating safe and effective exercise conditions for CKD patients is a pressing issue for controlling the introduction of dialysis and growth in medical expenditure. Hence, our future perspective is to explore how exercise under different exercise conditions affects kidneys in clinical applications, and this is very significant. The current study provides novel evidence that moderate‐intensity continuous exercise maintains RBF and does not induce increases in the biomarkers of renal injury. Our findings will provide basic data for the creation of effective exercise programs for preventing reductions in renal function, which could make a significant contribution to the development of appropriate medical care.

In conclusion, we investigated that the renal hemodynamic response to moderate‐intensity continuous exercise using ultrasound echo and revealed that a single bout of moderate‐intensity continuous exercise maintained RBF and estimated glomerular filtration rate and filtration fraction. Furthermore, a single bout of moderate‐intensity continuous exercise did not induce any significant increase in renal injury biomarkers.

## AUTHOR CONTRIBUTIONS

S. Kawakami, T.Y., S.K., and A.I. performed experiments; T.Y. and K.F. gathered the participants; S. Kawakami analyzed data; S. Kawakami, T.Y., and R.M. interpreted results of the experiments; S. Kawakami prepared figures; S. Kawakami drafted the manuscript; S. Kawakami, T.Y., and R.M. edited and revised the manuscript; S. Kawakami, T.Y., S.K., A.I., K.F., T.M., S.N., K.M., Y.U., Y.H., and R.M. approved the final version of the manuscript; S. Kawakami, T.Y., Y.H., and R.M. conceived and designed the research.

## FUNDING INFORMATION

This work was supported by the Japan Society for the Promotion of Science (JSPS) KAKENHI (grant numbers: 20 K23306 to S.K. and 19H04012 to Y.H.) and Fukuoka University Institute for Physical Activity, Fukuoka, Japan.

## CONFLICT OF INTEREST

No conflicts of interest, financial, or otherwise are declared by the authors.

## ETHICS STATEMENT

The study protocol in this study complied with the Declaration of Helsinki and the principles of Good Clinical Practice, and was approved by the Ethics Committee of Fukuoka University (No. 21‐02‐M1).

## References

[phy215420-bib-0001] Afsar, B. , Siriopol, D. , Aslan, G. , Eren, O. C. , Dagel, T. , Kilic, U. , Kanbay, A. , Burlacu, A. , Covic, A. , & Kanbay, M. (2018). The impact of exercise on physical function, cardiovascular outcomes and quality of life in chronic kidney disease patients: A systematic review. International Urology and Nephrology, 50, 885–904. 10.1007/s11255-018-1790-4 29344881

[phy215420-bib-0002] Bellos, I. , Pergialiotis, V. , & Kontzoglou, K. (2019). Renal resistive index as predictor of acute kidney injury after major surgery: A systematic review and meta‐analysis. Journal of Critical Care, 50, 36–43. 10.1016/j.jcrc.2018.11.001 30471559

[phy215420-bib-0003] Bongers, C. C. W. G. , Alsady, M. , Nijenhuis, T. , Hartman, Y. A. W. , Eijsvogels, T. M. H. , Deen, P. M. T. , & Hopman, M. T. E. (2017). Impact of acute versus repetitive moderate intensity endurance exercise on kidney injury markers. Physiological Reports, 5, e13544. 10.14814/phy2.13544 29263119 PMC5742704

[phy215420-bib-0004] Chapman, C. L. , Johnson, B. D. , Hostler, D. , Lema, P. C. , & Schlader, Z. J. (2020). Reliability and agreement of human renal and segmental artery hemodynamics measured using doppler ultrasound. Journal of Applied Physiology, 128, 627–636. 10.1152/japplphysiol.00813.2019 32027544

[phy215420-bib-0005] Clorius, J. , Mandelbaum, A. , Hupp, T. , Reinbold, F. , Zuna, I. , Denk, S. , Fellhauer, S. , & van Kaick, G. (1996). Exercise activates renal dysfunction in hypertension. American Journal of Hypertension, 9, 653–661. 10.1016/0895-7061(96)00036-2 8806977

[phy215420-bib-0006] Di Nicolò, P. , & Granata, A. (2017). Renal resistive index: Not only kidney. Clinical and Experimental Nephrology, 21, 359–366. 10.1007/s10157-016-1323-3 27530995

[phy215420-bib-0007] Eckerbom, P. , Hansell, P. , Cox, E. , Buchanan, C. , Weis, J. , Palm, F. , Francis, S. , & Liss, P. (2020). Circadian variation in renal blood flow and kidney function in healthy volunteers monitored with noninvasive magnetic resonance imaging. American Journal of Physiology. Renal Physiology, 319, F966–F978. 10.1152/ajprenal.00311.2020 33073586

[phy215420-bib-0008] Ene‐Iordache, B. , Perico, N. , Bikbov, B. , Carminati, S. , Remuzzi, A. , Perna, A. , Islam, N. , Bravo, R. F. , Aleckovic‐Halilovic, M. , Zou, H. , Zhang, L. , Gouda, Z. , Tchokhonelidze, I. , Abraham, G. , Mahdavi‐Mazdeh, M. , Gallieni, M. , Codreanu, I. , Togtokh, A. , Sharma, S. K. , … Remuzzi, G. (2016). Chronic kidney disease and cardiovascular risk in six regions of the world (ISN‐KDDC): A cross‐sectional study. The Lancet Global Health, 4, e307–e319. 10.1016/S2214-109X(16)00071-1 27102194

[phy215420-bib-0009] Go, A. S. , Chertow, G. M. , Fan, D. , McCulloch, C. E. , & Hsu, C. (2004). Chronic kidney disease and the risks of death, cardiovascular events, and hospitalization. The New England Journal of Medicine, 351, 1296–1305. 10.1056/NEJMoa041031 15385656

[phy215420-bib-0010] Grimby, G. (1965). Renal clearances during prolonged supine exercise at different loads. Journal of Applied Physiology, 20, 1294–1298. 10.1152/jappl.1965.20.6.1294 14257543

[phy215420-bib-0011] Hamano, K. , Nitta, A. , Ohtake, T. , & Kobayashi, S. (2008). Associations of renal vascular resistance with albuminuria and other macroangiopathy in type 2 diabetic patients. Diabetes Care, 31, 1853–1857. 10.2337/dc08-0168 18566339 PMC2518358

[phy215420-bib-0012] Heine, G. H. , Gerhart, M. K. , Ulrich, C. , Kaler, H. , & Girndt, M. (2005). Renal doppler resistance indices are associated with systemic atherosclerosis in kidney transplant recipients. Kidney International, 68, 878–885. 10.1111/j.1523-1755.2005.00470.x 16014069

[phy215420-bib-0013] Horio, M. , Imai, E. , Yasuda, Y. , Watanabe, T. , & Matsuo, S. (2013). GFR estimation using standardized serum cystatin C in Japan. American Journal of Kidney Diseases, 61, 197–203. 10.1053/j.ajkd.2012.07.007 22892396

[phy215420-bib-0014] Howden, E. J. , Fassett, R. G. , Isbel, N. M. , & Coombes, J. S. (2012). Exercise training in chronic kidney disease patients. Sport Medicine, 42, 473–488. 10.2165/11630800-000000000-00000 22587820

[phy215420-bib-0015] Juett, L. A. , James, L. J. , & Mears, S. A. (2020). Effects of exercise on acute kidney injury biomarkers and the potential influence of fluid intake. Annals of Nutrition & Metabolism, 76, 53–59. 10.1159/000515022 33774615

[phy215420-bib-0016] Kawakami, S. , Yasuno, T. , Matsuda, T. , Fujimi, K. , Ito, A. , Yoshimura, S. , Uehara, Y. , Tanaka, H. , Saito, T. , & Higaki, Y. (2018). Association between exercise intensity and renal blood flow evaluated using ultrasound echo. Clinical and Experimental Nephrology, 22, 1061–1068. 10.1007/s10157-018-1559-1 29525855

[phy215420-bib-0017] Kenney, W. L. , & Ho, C. W. (1995). Age alters regional distribution of blood flow during moderate‐intensity exercise. Journal of Applied Physiology, 79, 1112–1119. 10.1152/jappl.1995.79.4.1112 8567551

[phy215420-bib-0018] Kesaniemi, Y. K. , Danforth, E. , Jensen, M. D. , Kopelman, P. G. , & Lefèbvre, P. R. B. (2001). Dose‐response issues concerning physical activity and health: An evidence‐based symposium. Medicine and Science in Sports and Exercise, 33, S351–S358. 10.1097/00005768-200106001-00003 11427759

[phy215420-bib-0019] Khatir, D. S. , Pedersen, M. , Jespersen, B. , & Buus, N. H. (2015). Evaluation of renal blood flow and oxygenation in CKD using magnetic resonance imaging. American Journal of Kidney Diseases, 66, 402–411. 10.1053/j.ajkd.2014.11.022 25618188

[phy215420-bib-0020] Kosaki, K. , Kamijo‐Ikemori, A. , Sugaya, T. , Kumamoto, S. , Tanahashi, K. , Kumagai, H. , Kimura, K. , Shibagaki, Y. , & Maeda, S. (2020). Incremental short maximal exercise increases urinary liver‐type fatty acid‐binding protein in adults without CKD. Scandinavian Journal of Medicine & Science in Sports, 30, 709–715. 10.1111/sms.13618 31845418

[phy215420-bib-0021] Kotoku, K. , Yasuno, T. , Kawakami, S. , Fujimi, K. , Matsuda, T. , Nakashima, S. , Uehara, Y. , Tanaka, H. , Saito, T. , & Higaki, Y. (2019). Effect of exercise intensity on renal blood flow in patients with chronic kidney disease stage 2. Clinical and Experimental Nephrology, 23, 621–628. 10.1007/s10157-018-01685-3 30729347

[phy215420-bib-0022] Liguori, G. , Feito, Y. , Fountaine, C. , & Roy, B. (2021). ACSM's guidelines for exercise testing and prescription (11th ed.). Lippincott Williams & Wilkins.

[phy215420-bib-0023] Matsuo, S. , Imai, E. , Horio, M. , Yasuda, Y. , Tomita, K. , Nitta, K. , Yamagata, K. , Tomino, Y. , Yokoyama, H. , & Hishida, A. (2009). Revised equations for estimated GFR from serum creatinine in Japan. American Journal of Kidney Diseases, 53, 982–992. 10.1053/j.ajkd.2008.12.034 19339088

[phy215420-bib-0024] McDermott, B. P. , Smith, C. R. , Butts, C. L. , Caldwell, A. R. , Lee, E. C. , Vingren, J. L. , Munoz, C. X. , Kunces, L. J. , Williamson, K. , Ganio, M. S. , & Armstrong, L. E. (2018). Renal stress and kidney injury biomarkers in response to endurance cycling in the heat with and without ibuprofen. Journal of Science and Medicine in Sport, 21, 1180–1184. 10.1016/j.jsams.2018.05.003 29784554

[phy215420-bib-0025] Mehta, R. L. , Kellum, J. A. , Shah, S. V. , Molitoris, B. A. , Ronco, C. , Warnock, D. G. , & Levin, A. (2007). Acute kidney injury network: Report of an initiative to improve outcomes in acute kidney injury. Critical Care, 11, R31. 10.1186/cc5713 17331245 PMC2206446

[phy215420-bib-0026] Nakayama, M. , Sato, T. , Miyazaki, M. , Matsushima, M. , Sato, H. , Taguma, Y. , & Ito, S. (2011). Increased risk of cardiovascular events and mortality among non‐diabetic chronic kidney disease patients with hypertensive nephropathy: The Gonryo study. Hypertension Research, 34, 1106–1110. 10.1038/hr.2011.96 21796127

[phy215420-bib-0027] O'Neill, W. C. (2014). Renal resistive index. Hypertension, 64, 915–917. 10.1161/HYPERTENSIONAHA.114.04183 25156171

[phy215420-bib-0028] Pei, G. , Tang, Y. , Tan, L. , Tan, J. , Ge, L. , & Qin, W. (2019). Aerobic exercise in adults with chronic kidney disease (CKD): A meta‐analysis. International Urology and Nephrology, 51, 1787–1795. 10.1007/s11255-019-02234-x 31332699

[phy215420-bib-0029] Pennemans, V. , Rigo, J.‐M. , Faes, C. , Reynders, C. , Penders, J. , & Swennen, Q. (2013). Establishment of reference values for novel urinary biomarkers for renal damage in the healthy population: Are age and gender an issue? Clinical Chemistry and Laboratory Medicine, 51, 1795–1802. 10.1515/cclm-2013-0157 23648635

[phy215420-bib-0030] Poortmans, J. R. , Brauman, H. , Staroukine, M. , Verniory, A. , Decaestecker, C. , & Leclercq, R. (1988). Indirect evidence of glomerular/tubular mixed‐type postexercise proteinuria in healthy humans. American Journal of Physiology. Renal Physiology, 254, F277–F283. 10.1152/ajprenal.1988.254.2.F277 3125748

[phy215420-bib-0031] Poortmans, J. R. , Mathieu, N. , & de Plaen, P. (1996). Influence of running different distances on renal glomerular and tubular impairment in humans. European Journal of Applied Physiology and Occupational Physiology, 72, 522–527. 10.1007/BF00242285 8925826

[phy215420-bib-0032] Poortmans, J. R. , & Vanderstraeten, J. (1994). Kidney function during exercise in healthy and diseased humans. Sport Medicine, 18, 419–437. 10.2165/00007256-199418060-00006 7886356

[phy215420-bib-0033] Poussel, M. , Touzé, C. , Allado, E. , Frimat, L. , Hily, O. , Thilly, N. , Rousseau, H. , Vauthier, J.‐C. , & Chenuel, B. (2020). Ultramarathon and renal function: Does exercise‐induced acute kidney injury really exist in common conditions? Front Sport Act Living, 1, 1–7. 10.3389/fspor.2019.00071 PMC7739841 33344994

[phy215420-bib-0034] Rowe, G. C. , Safdar, A. , & Arany, Z. (2014). Running forward. Circulation, 129, 798–810. 10.1161/CIRCULATIONAHA.113.001590 24550551 PMC3981549

[phy215420-bib-0035] Santana, D. A. , Poortmans, J. R. , Dórea, E. L. , Machado, J. B. d. A. , Fernandes, A. L. , Sá‐Pinto, A. L. , Gualano, B. , & Roschel, H. (2017). Acute exercise does not impair renal function in nondialysis chronic kidney disease patients regardless of disease stage. American Journal of Physiology. Renal Physiology, 313, F547–F552. 10.1152/ajprenal.00131.2017 28515176

[phy215420-bib-0036] Schrezenmeier, E. , Barasch, J. , Budde, K. , Westhoff, T. , & Schmidt‐Ott, K. M. (2017). Biomarkers in acute kidney injury ‐ pathophysiological basis and clinical performance. Acta Physiologica, 219, 556–574. 10.1111/apha.12764 PMC5575831 27474473

[phy215420-bib-0037] Shimamoto, Y. , Kubo, T. , Tanabe, K. , Emori, H. , Katayama, Y. , Nishiguchi, T. , Taruya, A. , Kameyama, T. , Orii, M. , Yamano, T. , Kuroi, A. , Yamaguchi, T. , Takemoto, K. , Matsuo, Y. , Ino, Y. , Tanaka, A. , Hozumi, T. , Terada, M. , & Akasaka, T. (2017). Effects of intravenous bolus injection of nicorandil on renal artery flow velocity assessed by color doppler ultrasound. Journal of Cardiology, 69, 364–368. 10.1016/j.jjcc.2016.08.007 27613386

[phy215420-bib-0038] Svarstad, E. , Myking, O. , Ofstad, J. , & Iversen, B. M. (2002). Effect of light exercise on renal hemodynamics in patients with hypertension and chronic renal disease. Scandinavian Journal of Urology and Nephrology, 36, 464–472. 10.1080/003655902762467648 12623513

[phy215420-bib-0039] Thompson, S. , Wiebe, N. , Padwal, R. S. , Gyenes, G. , Headley, S. A. E. , Radhakrishnan, J. , & Graham, M. (2019). The effect of exercise on blood pressure in chronic kidney disease: A systematic review and meta‐analysis of randomized controlled trials. PLoS One, 14, e0211032. 10.1371/journal.pone.0211032 30726242 PMC6364898

[phy215420-bib-0040] Vanden Wyngaert, K. , Van Craenenbroeck, A. H. , Van Biesen, W. , Dhondt, A. , Tanghe, A. , Van Ginckel, A. , Celie, B. , & Calders, P. (2018). The effects of aerobic exercise on eGFR, blood pressure and VO2peak in patients with chronic kidney disease stages 3‐4: A systematic review and meta‐analysis. PLoS One, 13, e0203662. 10.1371/journal.pone.0203662 30204785 PMC6133282

[phy215420-bib-0041] Wołyniec, W. , Ratkowski, W. , Renke, J. , & Renke, M. (2020). Changes in novel AKI biomarkers after exercise. A systematic review. International Journal of Molecular Sciences, 21, 5673. 10.3390/ijms21165673 PMC7461060 32784748

[phy215420-bib-0042] Wołyniec, W. , Ratkowski, W. , Urbański, R. , Bartoszewicz, M. , Siluk, D. , Wołyniec, Z. , Kasprowicz, K. , Zorena, K. , & Renke, M. (2018). Urinary kidney injury molecule‐1 but not urinary neutrophil gelatinase associated lipocalin is increased after short maximal exercise. Nephron, 138, 29–34. 10.1159/000481179 28988230

[phy215420-bib-0043] Yamanaka, M. , Sugimoto, H. , Yokoyama, H. , Mochizuki, Y. , & Taniguchi, K. (2022). The renal artery pulsatility index enables real‐time monitoring of acute kidney injury after digestive surgery. Surgery, 171, 1406–1411. 10.1016/j.surg.2021.09.002 35094875

[phy215420-bib-0044] Yang, L. , Wu, X. , Wang, Y. , Wang, C. , Hu, R. , & Wu, Y. (2020). Effects of exercise training on proteinuria in adult patients with chronic kidney disease: A systematic review and meta‐analysis. BMC Nephrology, 21, 172. 10.1186/s12882-020-01816-7 32393200 PMC7216591

[phy215420-bib-0045] Zambraski, E. J. , Bober, M. C. , Goldstein, J. E. , Lakas, C. S. , & Shepard, M. D. (1981). Changes in renal cortical sialic acids and colloidal iron staining associated with exercise. Med Sci Sport Exerc, 13, 229–232. 10.1249/00005768-198104000-00004 6168885

